# Diseases of Children

**Published:** 1893-02

**Authors:** 


DISEASES OF CHILDREN.—A Manual for Students and Practitioners; by C. Alexander Rhodes, M. D., Instructor in Diseases of Children, New York Post-Graduate Medical College. Philadelphia, Lea Brothers & Co. Price $1.00.

THE author states that "the student and practitioner will fully appreciate that the intent of this compend is simply to present a summary of the diseases of children, and, therefore, its use is recommended only after a careful reading of the standard books from which its subject matter has been taken." Much credit is due Dr. Rhodes for the admirable manner in which the different subjects have been treated.

